# Sound transmission using a tuning fork for the diagnosis of developmental dysplasia of the hip in newborns

**DOI:** 10.3389/fped.2024.1397697

**Published:** 2024-10-28

**Authors:** Nicolás Padilla-Raygoza, Jéssica Plascencia-Roldán, Guadalupe Irazú Morales-Reyes, Luis Guillermo Patiño-Gutiérrez, Ariana Valeria Girón-Soto, Evaristo Antonio Meza-Galván, Itzel Marcela Anguiano-Canchola, Sergio Emmanuel Luna Santillana, José Juan Torres-Hernández, Gilberto Flores-Vargas

**Affiliations:** ^1^ Department of Research and Technological Development, Directorate of Teaching and Research, Institute of Public Health from Guanajuato State, Guanajuato, Mexico; ^2^ Division of Health Sciences, Campus Leon, University of Guanajuato, León, Mexico; ^3^ School of Medicine, University of Quetzalcoatl in Irapuato, Irapuato, Mexico; ^4^ Service of Pediatrics, Hospital General Guanajuato, Institute of Public Health from Guanajuato State, Guanajuato, Mexico; ^5^ Service of Radiology, Hospital General Guanajuato, Institute of Public Health from Guanajuato State, Guanajuato, Mexico; ^6^ Service of Surgery, Hospital General Guanajuato, Institute of Public Health from Guanajuato State, Guanajuato, Mexico

**Keywords:** newborns, hip, dysplasia, sound transmission, tuning fork, clinical diagnosis

## Abstract

**Introduction:**

This study aims to estimate the validity and reliability of sound transmission tests using a tuning fork and stethoscope compared with the usual clinical procedures for the diagnosis of developmental dysplasia of the hip in newborns from the state of Guanajuato, Mexico.

**Methodology:**

This is a cross-sectional study of newborns at the Guanajuato General Hospital of the Institute of Public Health from Guanajuato. The population under study comprised newborns born from April to May 2023. All parents of newborns were invited to participate. The Ortolani, Barlow, Peter–Baden, and sound transmission tests were performed with a tuning fork and stethoscope three times by two observers. Ultrasonography using the Graf technique was also applied to both hips. We evaluated the validity and repeatability of clinical procedures and sound transmission tests against ultrasonography as the gold standard. We calculated sensitivity, specificity, and predictive values for validity and Cohen's kappa for repeatability.

**Results:**

The sample consisted of 100 newborns (56% male and 44% female); 65% born by vaginal delivery. Sound transmission test results for sensitivity, specificity, the positive predictive value, and the negative predictive value were 87.88%, 95.81%, 80%, and 97.53%, respectively. The intra-observer kappa was 0.89 (95% CI = 0.80–0.97) and the inter-observer kappa was 0.85 (95% CI = 0.73–0.97). The validity and repeatability of the Ortolani, Barlow, and Peter–Baden tests were low compared with the sound transmission test.

**Conclusion:**

The sound transmission test using a tuning fork and stethoscope is helpful for the diagnosis of developmental dysplasia of the hip.

## 1 Introduction

Developmental dysplasia disease of the hip (DDDH) consists of hip alterations, ranging from a slight incongruence between the articular surfaces to the dislocation of the femoral head outside the acetabulum (1). DDDH originates from the interaction between the cartilage maturation, bony elements of the acetabulum, and the pressure applied by muscular forces on the femoral head (2).

In the USA, DDDH occurs in 1 in 100 live births in the form of instability and in 1 in 1,000 live births in the form of hip dislocation (3). In Mexico, 1% of neonates have DDDH, and 75% of macrosomia infants at birth have ultrasonographic evidence of hip abnormalities. Nevertheless, only 1 in every 7,000 live births progresses to hip dislocation (4). Fernandez (5) pointed out that 17% of affected children were diagnosed by the medical doctor and the rest by the family, generally in the second semester of the infant's life, when the infant started walking.

The cause of DDDH may include maternal hormones, macrosomia, breech presentation with extension of the lower extremities, inadequate obstetric practices during birth and in postnatal life, and improper wrapping or carrying techniques for the baby (6, 7). DDDH is classified into physiological immaturity, subluxation, luxation, and dislocation of the hip. The clinical diagnosis is established through procedures that should be routinely performed during the examination of neonates and infants: Ortolani, Barlow, Peter–Baden, and limitation of abduction, among others (1). These procedures only detect hips with subluxation, luxation, or dislocation (1, 6, 7), and physiological immaturity may go undiagnosed, potentially becoming a risk factor for progression to subluxation, luxation, or dislocation.

In 1987, Stone et al. (8) described sound transmission testing with a tuning fork and stethoscope for clinical diagnosis, which was later replicated by Padilla-Raygoza and Figueroa-Ferrari (9) in Mexico, utilizing the properties of bone to transmit sound. The definitive diagnosis is made in neonates and infants up to 3 months of age, using the Graf technique in ultrasonography. Radiographs are not recommended in children under 8–12 weeks of postnatal life due to the incomplete ossification of the femoral head. This study aimed to compare the validity and reliability of sound transmission tests with a tuning fork and stethoscope with the usual clinical procedures for the diagnosis of DDDH in neonates from Guanajuato, Mexico.

## 2 Participants and methods

The protocol was approved by the Ethics Committee for Research of Hospital General Salamanca (registry number CEI-HGS007-2023) on 13 March 2023.

### 2.1 Study design

This is a quantitative, cohort, longitudinal, prospective, prolective, and analytical study. The follow-up period for each newborn was 28 days. The sound transmission and clinical procedures were performed 4–6 days after the delivery, and the hip ultrasonography was carried out 2–4 weeks after the sound transmission and clinical procedures. The universe of this study consisted of newborns born at the Guanajuato General Hospital (GGH) from April to May, 2023. The parents of all neonates were invited to participate. No sampling method was used. Progressively, starting on 1 April and continuing until the recruitment of 100 neonates, the parents were invited to have their newborns participate in the study.

### 2.2 Sample size calculation

The minimum sample size calculation is 100 neonates with DDDH, assuming a sensitivity value for the sound transmission of 90%, 95% precision, and 80% power (Epidat, 4.2, Xunta de Galicia, WHO, Universidad CES).

### 2.3 Selection criteria

The inclusion criteria were neonates born at the GGH whose parents consented to their participation by signing the informed consent form. The exclusion criteria were neonates with congenital rigid or embryological hip dislocation. The elimination criteria were neonates with incomplete evaluations due to not attending the hip ultrasound.

### 2.4 Variables

The sociodemographic variables included age, sex, gestational age based on the date of the mother's last menstrual period, and mode of delivery.

#### 2.4.1 Independent variables

The comparative sound transmission test (tuning fork) is a dichotomous categorical variable. It measures the transmission of the sound in the hip, from being in extension to being in flexion. An increase in sound when flexing the hip is considered with DDDH. If the sound remains the same or decreases, the individual is considered free of DDDH. Results are presented as frequencies and percentages.

The Ortolani test is a dichotomous categorical variable. It refers to the clinical maneuver evaluating both hips simultaneously to detect a clicking sound in one of them. It is measured as “yes” or “no” and is presented as frequencies and percentages.

The Barlow test is a dichotomous categorical variable. It is a clinical maneuver that evaluates each hip separately, looking for a snapping sound. It is measured as “yes” or “no” and is presented as frequencies and percentages.

The Peter–Baden test is a dichotomous categorical variable. It is a clinical maneuver that evaluates asymmetry in the groin, thigh, and gluteal folds. It is considered positive if asymmetry is present and negative if it is not. Results are presented as frequencies and percentages.

#### 2.4.2 Dependent variable

The ultrasonographic evaluation result is an ordinal categorical variable. It refers to the alteration of the hip joint, measured with hip ultrasonography using the Graf technique, which consists of a dynamic test and a static test, measuring the alpha and beta angles of the hips. A healthy hip is classified as Graf I (*α* > 60° and *β* < 55°), physiological immaturity is classified as Graf II (*α* = 44°–59° and *β* = 55°–77°), and subluxation, luxation, or dislocation are classified as Graf III or IV (*α* < 43° and *β* > 77°) (10, 11).

As procedure, when receiving a pregnant woman for obstetric resolution by the GGH, the program objectives and the risks and benefits to the neonate of participating were explained. After the birth of the newborn, either the mother or the father were asked to sign the informed consent form. If they signed it, the sound transmission test was applied. It consisted of placing the neonate in dorsal recumbency with the lower extremities extended, the 256 cycles/s tuning fork vibrating on the patella, and the diaphragm of the stethoscope on the pubis and perceiving the sound. The hip must be flexed to 90° to perceive the sound. The usual clinical procedures were also applied on three occasions: the first two by the pediatrician of the service and a third by the pediatric residents. The neonates were also scheduled for a hip ultrasound using the Graf technique.

### 2.5 Statistical analysis

For the validity of the sound transmission test using a tuning fork and the clinical procedures (Ortolani, Barlow, Peter–Baden, and abduction limitation), sensitivity, specificity, and predictive values were calculated using hip ultrasound with the Graf technique as the gold standard. Cohen's kappa was calculated for intra-observer reliability (comparing the first and second measurements by the pediatrician) and inter-observer reliability (comparing the pediatrician's measurements with those of the pediatric resident) to assess the repeatability of the sound transmission test. Statistical analysis was performed using STATA 13.0® (StataCorp., College Station, TX, USA).

## 3 Results

The sample size was 100. Table 1 presents their sociodemographic characteristics. Males neonates comprised 56% of the sample and female neonates 44%; 65% of the all newborns were born via vaginal delivery. The average age at first contact after birth was 0.79 ± 0.71 days, and the average gestational age based on the last menstrual period was 39.22 ± 1.55 weeks.

**Table 1:** Distribution of sociodemographic variables of the participating neonates (*n* = 100).

Categorical variables	*n*	%
Sex		
Male	56	56.00
Female	44	44.00
Delivery method		
Vaginal	65	65.00
Cesarean procedure	35	35.00
Quantitative variables	Range	Mean ± standard deviation
Age (days)	0.13–4	0.79 ± 0.71
Gestational age LMP (weeks)	35–43.2	39.22 ± 1.55

LMP, last menstrual period.

Source: Research files.

According to the hip ultrasound using the Graf technique, the results predominantly showed physiological immaturity: 13% in the right hip and 17% in the left hip. Only two cases of subluxation and two cases of hip dislocation were detected (Table 2).

**Table 2:** Diagnosis of hip with ultrasound using the Graf technique.

	Left hip		Right hip	
	*n*	%	*n*	%
Graf technique				
I (without alterations)	82	82.00	85	85.00
II physiologic immaturity	17	17.00	13	13.00
III subluxation	0	0.00	1	1.00
IV luxation	1	1.00	1	1.00

Source: Research files.

When comparing the three assessments using sound transmission and hip ultrasound with the Graf technique, the sensitivity was similar, at over 80%, specificity exceeded 90%, the positive predictive value ranged between 70% and 80%, and the negative predictive value was above 90% (Table 3).

**Table 3:** Validity of sound transmission with a tuning fork and stethoscope.

	US positive	US negative	Sensitivity (%)	Specificity (%)	Positive predictive value (%)	Negative predictive value (%)
First measurement of sound transmission			84.85	95.81	80.00	96.97
Positive	28	7				
Negative	5	160				
Second measurement of sound transmission			87.88	93.41	72.50	97.50
Positive	29	11				
Negative	4	156				
Third measurement of sound transmission			87.88	94.61	76.32	97.53
Positive	29	9				
Negative	4	158				

US, ultrasonography.

Source: Research files.

The Ortolani procedure is used to diagnose subluxated or dislocated hips; however, it does not detect dysplasia. As shown in Table 4, the sensitivity was below 20% and the positive predictive value was under 50%.

**Table 4:** Validity of the Ortolani maneuver.

	US positive	US negative	Sensitivity (%)	Specificity (%)	Positive predictive value (%)	Negative predictive value (%)
The first measurement of Ortolani			12.12	94.61	30.77	84.49
Positive	4	9				
Negative	29	158				
The second measurement of Ortolani			15.15	95.81	41.67	85.11
Positive	5	7				
Negative	28	160				
The third measurement of Ortolani			12.12	93.41	25.67	84.32
Positive	4	11				
Negative	29	156				

US, ultrasonography.

Source: Research files.

For the Barlow maneuver, a similar issue arises as with the Ortolani maneuver. As it does not detect hip dysplasia and only identifies subluxated or dislocated hips, the sensitivity and positive predictive value were very low across the three measurements (Table 5).

**Table 5:** Validity of the Barlow maneuver.

	US positive	US negative	Sensitivity (%)	Specificity (%)	Positive predictive value (%)	Negative predictive value (%)
The first measurement of Barlow			12.12	95.81	36.36	89.42
Positive	4	7				
Negative	29	160				
The second measurement of Barlow			18.18	95.81	46.15	85.56
Positive	6	7				
Negative	27	160				
The third measurement of Barlow			15.15	94.61	35.71	84.95
Positive	5	9				
Negative	28	158				

US, ultrasonography.

Source: Research files.

Fold asymmetry is a sign that raises suspicion of subluxation or dislocation of the hip. However, there is no evidence that it indicates hip dysplasia, as demonstrated by the very low sensitivities (<10%) and positive predictive values of 50% or less (Table 6).

**Table 6:** Validity of fold asymmetry or the Peter–Baden sign.

	US positive	US negative	Sensitivity (%)	Specificity (%)	Positive predictive value (%)	Negative predictive value (%)
The first measurement of Peter–Baden			6.06	98.80	50.00	85.05
Positive	2	2				
Negative	31	165				
The second measurement of Peter–Baden			6.06	98.20	40.00	84.10
Positive	2	3				
Negative	31	164				
The third measurement of Peter–Baden			0	97.60	0	83.16
Positive	0	4				
Negative	33	163				

US, ultrasonography.

Source: Research files.

The intra-observer reliability was *K* = 0.89 and the inter-observer reliability was *K* = 0.85, indicating that the sound transmission test measurements were applied similarly by both the pediatrician and the pediatric resident (Table 7).

**Table 7:** Reliability of sound transmission.

	The first measurement of sound transmission positive	The first measurement of sound transmission negative	Kappa	95% CI
The second measurement of sound transmission			0.89	0.80–0.97
Positive	34	6		
Negative	1	159		
The third measurement of sound transmission			0.85	0.73–0.95
Positive	32	6		
Negative	3	159		

95% CI, 95% confidence interval.

Source: Research files.

The intra-observer reliability of the Ortolani maneuver was 0.70, but the inter-observer reliability was much lower (0.54) (Table 8).

**Table 8:** Reliability of the Ortolani maneuver.

	The first measurement of Ortolani positive	The first measurement of Ortolani negative	Kappa	95% CI
The second measurement of Ortolani			0.70	0.49–0.91
Positive	9	3		
Negative	4	184		
The third measurement of Ortolani			0.54	0.31–0.77
Positive	8	7		
Negative	5	180		

95% CI, 95% confidence interval.

Source: Research files.

The Barlow maneuver showed a good intra-observer reliability of 0.80 (measurements made by the pediatrician), but the inter-observer reliability decreased to 0.62 (Table 9). Fold asymmetry showed an intra-observer reliability of 0.89. A comparison of the measurement by the pediatrician with that of the pediatric resident yielded a Kappa of 0.49 (Table 10).

**Table 9:** Reliability of the Barlow maneuver.

	The first measurement of Barlow positive	The first measurement of Barlow negative	Kappa	95% CI
The second measurement of Barlow			0.82	0.65–0.99
Positive	10	3		
Negative	1	186		
The third measurement of Barlow			0.62	0.39–0.85
Positive	8	6		
Negative	3	183		

95% CI, 95% confidence interval.

Source: Research files.

**Table 10:** Reliability of fold asymmetry or the Peter–Baden sign.

	The first measurement of Peter–Baden positive	The first measurement of Peter–Baden negative	Kappa	95% CI
The second measurement of Peter–Baden			0.89	0.67–1.00
Positive	4	1		
Negative	0	195		
The third measurement of Peter–Baden			0.49	0.06–0.92
Positive	2	2		
Negative	2	194		

95% CI, 95% confidence interval.

Source: Research files.

## 4 Discussion

In the sample of 100 neonates, 33 abnormal hips were detected (15 right hips and 18 left hips) (Table 2). It is important to note that, among the 100 newborns, 13 cases of right physiological immaturity and 17 cases of left subluxation were detected, along with 1 case of right subluxation, 1 case of right dislocation, and 1 case of left dislocation. Dislocation has been reported to occur in 1:1,000 births (3). The 30% incidence of physiological immaturity observed is similar to the rates reported in Mexico (7). In the USA, the prevalence of immaturity is 1% (3).

The sample for the present report consisted of newborns from a public hospital in Guanajuato City. The samples from other studies on the transmission of sound in the state of Guanajuato presented the following characteristics. In the first study in Celaya (9), the sample comprised 104 newborns and children aged up to 2 years from private healthcare institutions. The gold standard was the AP radiography in the neutral position and abduction. The results included 32 true positives (30.77%) and 11 false positives. The second sample comprised 150 newborns and children aged up to 28 days (7). In this case, the participants came from a public institution in Celaya. The gold standard was the Graf technique applied to hip ultrasounds. The results included 54% of the cases with physiological immaturity, 4.6% with subluxation, and none with luxation.

Regarding the sociodemographic variables across the mentioned samples, there were no differences between the mentioned samples. In addition, the frequencies of physiological immaturity, subluxation, or luxation were similar.

The sound transmission test showed superior validity and reliability than standard clinical procedures. Stone et al. (8) did not calculate the validity of the sound transmission test. Nevertheless, based on the published data, a sensitivity value of 72% and specificity of 88% can be computed, which are lower than those obtained in this research (Table 2). Validity demonstrates that the sound transmission should be measured for alterations of the hip, compared with the gold standard. In children up to 2 years of age from Celaya, the sensitivity was 74.4% and the specificity was 96.9% (9), which were slightly different from those obtained in this sample (Table 2).

Padilla and Figueroa (7) obtained a sensitivity of 86.36%, a specificity of 87.09%, a negative predictive value of 90.47%, and a negative predictive value of 81.81%, applying the sound transmission test. In this study, the sensitivity was 84.85%–87.88% in the three measurements (Table 3).

The tuning fork reliability for the sound transmission test was greater than 0.80 for both intra- and inter-observer comparisons (Table 6). Reliability shows that the results are similar independently of who applies the test; a kappa higher than 0.8 is excellent reliability. Reliability remains high in different observers because it is a simple test.

The clinical procedures of Ortolani, Barlow, and fold asymmetry only detect subluxation or dislocation. As they do not detect dysplasia, the validity for DDDH is very low, as demonstrated in this study (Tables 4, 5, and 6) and reported by Padilla and Figueroa (7). Dysplasia is much more prevalent than subluxation or dislocation. The validity and reliability values are low as the clinical procedures included in the study do not detect dysplasia.

Concerning more objective measures, the change in logarithm based on 10 of decibels (log_10_dB), when comparing the transmission of sound with the extended vs. flexed legs, was measured using the electroacoustic probe. The difference in log_10d_B detected was from −1.4 (false positives) to 3.1 in hips without alterations. For those with physiological immaturity, the difference was from −2.8 to 1.4 (false negatives). Finally, for subluxation, the difference of log_10_dB was −1, and for luxation, it was −0.1 (12, 13). If dysplasia of the hip is not detected early, it can progress into subluxation or dislocation. Moreover, if children begin to walk while having this disease, they will need surgery on the hip.

### 4.1 Limitations

In this study, only the sound transmission test with extension or flexion was applied as it evaluates each hip separately, outperforming the comparative sound transmission test, which can yield false negatives in the case of bilateral DDDH because it compares the sound transmission in one hip with the other one (7–9).

Another limitation is that although Stone et al. (8) described the sound transmission tests in 1988, which were replicated in Mexico, we could not find articles on sound transmission for the DDDH diagnosis. Sound transmission tests using a tuning fork depend on the auditory accuracy of the observer, which could explain the differences in results between Stone et al. (8), Padilla and Figueroa (7), and this study.

## 5 Conclusion

Sound transmission with a tuning fork and stethoscope is a valuable tool for the diagnosis of DDDH in neonates. It aids in assessing the clinical suspicion of DDDH, which is confirmed through hip ultrasound using the Graf technique. The clinical procedures of Ortolani, Barlow, and Peter–Baden are effective at diagnosing subluxation or the dislocation of the hip. The sound transmission test serves as an additional tool for clinical diagnosis.

It is advisable to conduct studies with larger sample sizes. One of our future projects aims to include more newborns from the state of Guanajuato. This study demonstrated an optional, validated, and reliable tool for the diagnosis of DDDH in newborns.

## Data Availability

The original contributions presented in the study are included in the Supplementary Material, further inquiries can be directed to the corresponding author.

## References

[1] Cymet-Ramirez J Alvarez-Martinez MM Garcia-Pinto G Frias-Austria R Meza-Vernis A Rosales-Muñoz ME El diagnóstico oportuno de la displasia de cadera. Enfermedad discapacitante de por vida. Consenso del colegio mexicano de ortopedia y traumatología. Acta Ortop Pediátr. (2011) 25(5):313–22. 22509638

[2] Dezateux C Rosendahl K. Developmental dysplasia of the hip. Lancet. (2007) 369(9572):1541–52. 10.1016/S0140-6736(07)60710-7 17482986

[3] American Academy of Pediatrics, Committee on Quality Improvement, Subcommittee on Developmental Dysplasia of the Hip. Clinical practice guideline: early detection of developmental dysplasia of the hip. Pediatrics. (2000) 105: 896–905. 10.1542/peds.105.4.896 10742345

[4] Figueroa-Ferrari RC Padilla-Raygoza N. Congenital dislocation of the hip in the macrosomic newborn. Ultrasound aspects. Rev Med IMSS. (1994) 32:277–9.

[5] Fernandez E. Congenital hip dislocation: reduction with modified straps pavlik children one year of age. Rev Mex Ortop Traumatol. (1989) 3:30–4.

[6] Padilla N. Developmental dysplasia of the hip. In: Martinez R, editor. Martinez Health of Children and Adolescents. 7th ed. México City: El Manual Moderno (2013). p. 1513–17.

[7] Padilla N Figueroa RC. Sound transmission tests in the diagnosis of congenital dislocation of the hip in the neonate. Rev Mex Pediatr. (1996) 63:265–8.

[8] Stone MH Richardson JB Bennet JC. Another clinical test for congenital dislocation of the hip. Lancet. (1987) 1:954–5. 10.1016/S0140-6736(87)90296-0 2882344

[9] Padilla-Raygoza N Figueroa-Ferrari RC. Diagnosis of the hip luxation by sound compared transmission. Rev Mex Pediatr. (1992) 59(5):149–51.

[10] Arti H Mehdinasab SA Arti S. Comparing results of clinical versus ultrasonographic examination in developmental dysplasia of hip. J Res Med Sci. (2013) 18(12):1051–5. 24523795 PMC3908525

[11] Kowalczyk B Felus J Kwinta P. Developmental dysplasia of the hip: the problems in the diagnosis process in our own experience. Med Wieku Rozwoj. (2005) 9(3):395–406. 16547386

[12] Padilla-Raygoza N Olvera-Villanueva G Delgado-Sandoval MC Córdova-Fraga T Sosa-Aquino MA Beltrán-Campos V. Validity and reliability of electroacoustic probe for diagnosis of developmental dysplasia of the hip. BMC Pediatr. (2017) 17:149. 10.1186/s12887-017-0903-z 28629337 PMC5477343

[13] Padilla-Raygoza N. Differences on sound transmission with electroacoustic probe. (2023) (Supplementary Material).

